# Dual Delivery of Cells and Bioactive Molecules for Wound Healing Applications

**DOI:** 10.3390/molecules30071577

**Published:** 2025-03-31

**Authors:** Petras Winkler, Yong Mao

**Affiliations:** Department of Chemistry and Chemical Biology, Rutgers University, Piscataway, NJ 08854, USA; pjw103@scarletmail.rutgers.edu

**Keywords:** wound healing, GelMA microparticles, hydrogel, human mesenchymal stem cells, delivery, cell carriers, nanoparticles, antibiotics

## Abstract

Chronic wounds not only cause significant patient morbidity but also impose a substantial economic burden on the healthcare system. The primary barriers to wound healing include a deficiency of key modulatory factors needed to progress beyond the stalled inflammatory phase and an increased susceptibility to infections. While antimicrobial agents have traditionally been used to treat infections, stem cells have recently emerged as a promising therapy due to their regenerative properties, including the secretion of cytokines and immunomodulators that support wound healing. This study aims to develop an advanced dual-delivery system integrating stem cells and antibiotics. Stem cells have previously been delivered by encapsulation in gelatin methacrylate (GelMA) hydrogels. To explore a more effective delivery method, GelMA was processed into microparticles (MP). Compared to a bulk GelMA hydrogel (HG) encapsulation, GelMA MP supported greater cell growth and enhanced in vitro wound healing activity of human mesenchymal stem cells (hMSCs), likely due to a larger surface area for cell attachment and improved nutrient exchange. To incorporate antimicrobial properties, the broad-spectrum antibiotics penicillin/streptomycin (PS) were loaded into a bulk GelMA hydrogel, which was then cryo-milled into MPs to serve as carriers for hMSCs. To achieve a more sustained antibiotic release, gelatin nanoparticles (NP) were used as carriers for PS. PS was either incorporated during NP synthesis (NP+PS(S)) or absorbed into NP after synthesis (NP+PS(A)). MPs containing PS, NP+PS(S), or NP+PS(A) were tested for their cell carrier functions and antibacterial activities. The incorporation of PS did not compromise the cell-carrying function of MP configurations. The anti-*S. aureus* activity was detected in conditioned media from MPs for up to eight days—four days longer than from bulk HG containing PS. Notably, the presence of hMSCs prolonged the antimicrobial activity of MPs, suggesting a synergistic effect between stem cells and antibiotics. PS loaded via synthesis (NP+PS(S)) exhibited a delayed initial release, whereas PS loaded via absorption (NP+PS(A)) provided a more immediate release, with potential for sustained delivery. This study demonstrates the feasibility of a dual-delivery system integrating theraeutic hMSCs with tunable antibiotic release. This approach offers a multifaceted wound treatment strategy and underlines the potential for co-delivering bioactive molecules alongside cells, with applications extending beyond wound healing.

## 1. Introduction

Chronic wounds are currently a major health issue affecting 2.5% of the United States population in 2020 [1]. These wounds are problematic since they remain in the inflammatory phase of healing in part due to a lack of growth factors and/or infection. As a result, they do not close. Exposure to the outside environment increases microbial infection rates and biofilm formation, further inhibiting wound closure and heightening the probability of life-threatening conditions such as sepsis. Of the numerous dangerous pathogens, *S. aureus* is the most prominent, and it infects approximately twenty percent of individuals with diabetic foot ulcers in North America [2]. Up to 30% of these infections will result in death [3]. Last resort treatment often requires amputation of the limb, and diabetic foot ulcers alone are responsible for 150,000 amputations in one year [4]. Even if treated, serious cases of infection also leave patients more prone to other health complications such as musculoskeletal disease, endocrine disease, and cancer [5].

Treatment of these wounds is complex and can require several therapeutic approaches. Current clinical methods to treat chronic wounds consist of antibiotic ointments, gels, or physical coverings such as bandages [6]. While these stave off infection, they do not cure the underlying issue and must be reapplied regularly. Some surgical methods aimed at stimulating wound closure include skin grafts, negative pressure therapy, and debridement [7]. Negative pressure therapy removes exudate that could potentially become infected while also promoting the formation of granulation tissue, but evidence to support its ability to prevent infection has been conflictual [8]. Skin grafts can be fragile, and synthetic alternatives tend to be very costly [9]. Debridement involves removing compromised tissue that has a diminished ability to migrate to close the wound, but it still needs to be paired with other methods to bolster healing [10]. Several research groups have studied the effects of the topical application of growth factors on wound healing [11], but chronic wounds are incredibly complex and likely require more than one factor to treat them most effectively [12]. In addition, neutrophils in the wound environment secrete enzymes that degrade these growth factors [13]. Given the myriads of interactions between growth factors and the difficulty of exogenously replicating them, interest in alternative approaches has arisen.

Stem cell therapies are a promising method to aid in the healing of chronic wounds. Their ability to produce and disseminate growth factors such as VEGF [14], FGF [15], and PDGF [16] helps stimulate the wound healing process. In comparison to exogenous growth factors, cells transferred to the wound environment can consistently release these molecules rather than requiring repeated applications [17]. Mesenchymal stem cells (MSCs) injected directly into the wound site have been shown to drastically aid in the closure of diabetic foot ulcers [18]. However, the direct addition of cells to the wound site does not maximize their potential, as they have a very low survival and retention rate [19]. To rectify this attrition, scaffolds must be provided for the cells to adhere to. The use of self-assembled protein nanofibers or synthetic amino acid aggregates as scaffold materials has resulted in enhanced fibroblast cellular function [20,21]. Cells can also be encapsulated in hydrogels, which are malleable scaffolds that aid in cell growth and can be modified to release various compounds to stimulate wound closure. They can be synthesized using synthetic or natural polymers, but one promising material is gelatin methacrylate (GelMA). GelMA is formed by functionalizing gelatin with methacrylic anhydride, which helps bolster the versatility of the material. Since it is derived from collagen, the most abundant protein in the body and the main component of the extracellular matrix, it is a highly biocompatible material and also provides an excellent stratum for cells to adhere [22].

However, hydrogels do have two main shortcomings. The first is that embedded hydrophilic molecules tend to diffuse into the surroundings quickly, leading to a high initial dose and little to no prolonged effect, which is known as “burst release” [23]. As a result, they cannot effectively deliver molecules that require consistent levels to remain effective, such as antibiotics, to prevent infections. Cai et al. successfully created a vancomycin-loaded hydrogel composed of porcine extracellular matrix (ECM), which is a network of proteins that support cellular adhesion and is primarily composed of collagen. They demonstrated that their scaffold could inhibit the growth of *S. aureus.* However, the design is limited since the released vancomycin was only detectable for up to nine hours [24]. While this helps stave off initial infection, it is insufficient for chronic wounds constantly exposed to the environment. One promising method of prolonging drug release is to load molecules within nanoparticles. Yu et al. were able to load curcumin within PGA nanoparticles and embed these into a gelatin–genipin hydrogel. They observed that the composite hydrogel–nanoparticle structure reduced the release of curcumin to approximately 60% after 24 h compared to the gel containing free curcumin [25].

The second shortcoming hydrogels face is that cells prefer to grow on the outer edges and surface of the scaffold since these areas can quickly uptake nutrients and release unnecessary metabolic byproducts. Cells encapsulated in the center of the hydrogel experience reduced growth and biological activity. An ideal scaffold would maintain the same biocompatibility and cell adhesive properties as hydrogels while increasing the surface area. As a result, microcarriers have become the focus of attention. One study published by Dr. Ozhava et al. compared the viability of cells seeded on microspheres and cells encapsulated in a GelMA hydrogel. Not only did the cells on the microspheres have a higher capacity for proliferation, but they also were able to stimulate wound closure of human dermal fibroblast and the production of PDGF-β by human dermal microvascular endothelial cells in vitro [16]. The shape of the microcarrier can be further refined since spheres exhibit the lowest surface-area-to-volume ratio of any three-dimensional shape [26].

Our study investigates GelMA microparticles (MP) as possible carriers for cells to the wound environment, as well as methods to functionalize them with antibiotic properties. Microparticles were created via cryo-milling of GelMA hydrogels, which created irregular shapes to increase surface area. Human bone marrow-derived mesenchymal stem cells (hMSCs) were seeded on MPs and compared to cells encapsulated in GelMA hydrogels to determine if the irregular topology was able to bolster cell growth and growth factor production. Cells grown on microparticles were shown to have higher cell viability through DNA quantification and a better ability to stimulate human dermal fibroblast (HDF) migration in a scratch wound assay. To impart a dual carrier function to MP, penicillin-streptomycin (PS) was loaded into MP for its antimicrobial properties. To further enhance the sustained release of antimicrobial activity for wound applications, gelatin nanoparticles (NP) were employed as carriers for PS. PS was either entrapped in NP via a double desolvation synthesis method or loaded through absorption. The anti-*S. aureus* activity of MP in various configurations was evaluated and compared. In the absence of cells, PS delivered via MPs or bulk hydrogel (HG) showed antimicrobial effects. However, when MPs or HG carried both cells and PS, the anti-*S. aureus* activity was prolonged. Compared to HG, MPs demonstrated greater wound healing potential and a more sustained release of antimicrobial molecules. Among MP configurations, NP loaded with PS via absorption provided a more sustained release of anti-*S. aureus* activity within a potentially therapeutically relevant window. These promising results warrant further evaluation of this MP dual-delivery system for wound healing applications.

## 2. Results and Discussion

### 2.1. Preparation of Gelatin Methacrylate (GelMA) Based Cell Carriers

Bulk GelMA hydrogels are widely used as cell carriers due to their biocompatibility and mild crosslinking conditions [27]. In this approach, cells are mixed with a GelMA solution and crosslinked under a 405 nm light using a photoinitiator [28]. While cells encapsulated in the hydrogel remain viable and can be delivered to targeted sites, their viability and functionality are limited by physical constraints and restricted nutrient-waste exchange within the bulk hydrogel [29].

To address these limitations, hydrogel microparticles have been explored to increase surface area and enhance cell-nutrient accessibility [30]. Hydrogel microspheres, such as gelatin microspheres, have demonstrated improved support for cell growth and function [16]. GelMA microspheres can be generated using microfluidic systems with precisely controlled crosslinking [31]. Alternatively, large-scale production is possible through an oil-water emulsion method combined with high-temperature crosslinking [32]. Since spheres have the smallest surface-area-to-volume ratio among all shapes [26], we aimed to create microparticles with irregular shapes to maximize surface area.

**Figure 1:**
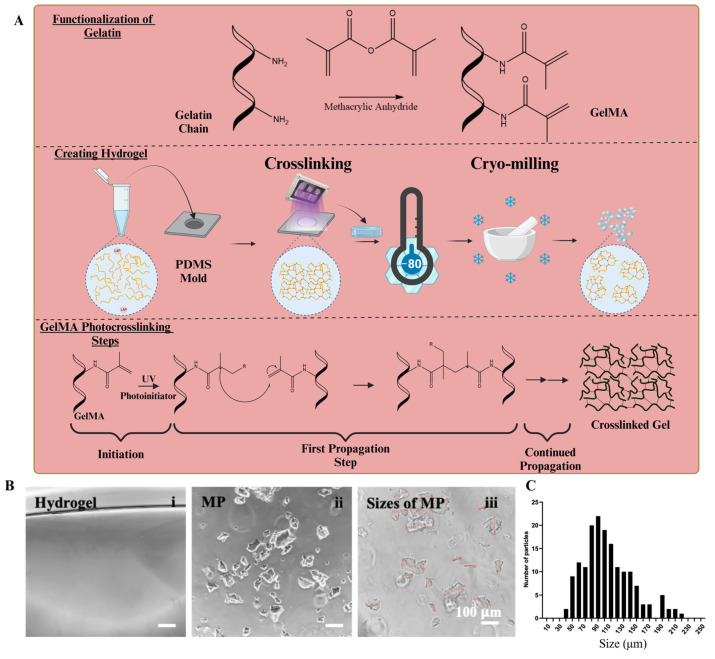
Making of GelMA microparticles (MP). (**A**) The key steps of making GelMA MP are illustrated. (**B**) Bulk hydrogel before cryo-milling (i), after milling (ii), and measuring of the sizes of MP (iii) using ECHO microscope and imaging software. Scale bar = 100 µm. (**C**) The size distribution of MP (Average Size = 112.4 ± 64.5 µm).

To fabricate GelMA microparticles (MP), bulk GelMA hydrogels were first formed by UV crosslinking a 10% GelMA solution in a designated mold (Figure 1A), which causes a chain reaction covalently binding the GelMA chains together [33]. The resulting bulk hydrogel (Figure 1B(i)) was then frozen at −80 °C or in liquid nitrogen before micronization using a mortar and pestle. The particle size distribution was monitored under a light microscope, and the grinding process was repeated twice to achieve the desired size range. Previous studies suggest that human mesenchymal stem cells (hMSCs) prefer gelatin microspheres with diameters ≤100 μm [16]. Thus, we targeted a similar size range in this study.

The resulting MP exhibited diverse shapes and sizes. When their longitudinal lengths were measured (Figure 1B(iii)), the size distributions followed a mild-to-moderate right-skewed distribution when plotted on a logarithmic scale, peaking at approximately 100 μm (Figure 1C). The average microparticle size used in this study was 112.4 ± 64.5 μm. The hydrogel MPs within the desired size range were then collected and lyophilized.

### 2.2. Culturing Human Mesenchymal Stem Cells on GelMA Microparticles and in GelMA Hydrogel

**Figure 2:**
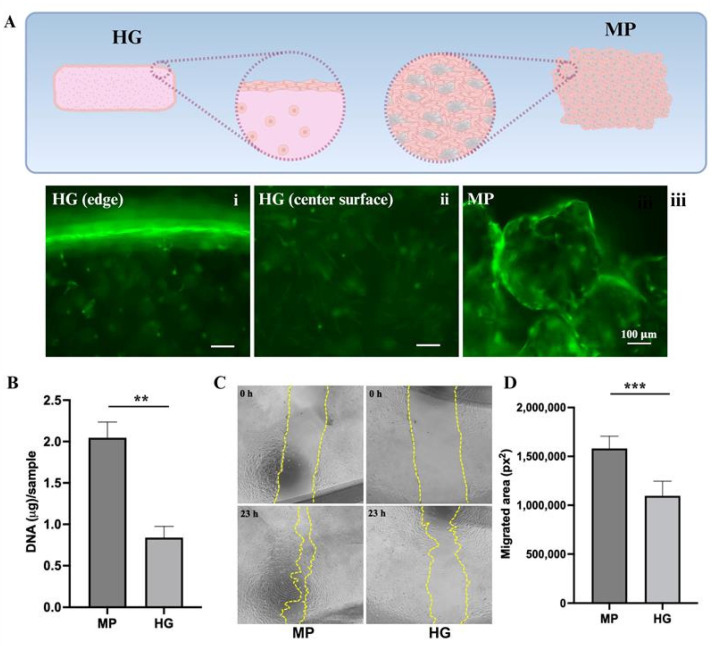
Viability and functionality of hMSCs on MP and HG. (**A**) hMSCs cultured in HG or on MP for 7 days, followed by CalceinAM staining. Representative images were shown. Scale bar = 100 µm. (**B**) Quantification of total DNA isolated from cells cultured on MP or HG. (**C**) Representative images of the wound areas of HDF at 0 h and 23 h. The edges of the wound are traced by the dotted yellow lines. (**D**) The migrated areas were measured using ImageJ software. Migrated area = Area_0h_ − Area_23h_. Data shown are mean ± SD (** *p*-value < 0.01, *** *p*-value < 0.005).

To evaluate and compare the growth of human mesenchymal stem cells (hMSCs) on GelMA microparticles (MP) and within bulk GelMA hydrogel (HG), 6 × 10^4^ hMSCs per sample were seeded onto either rehydrated MP (3 mg dry weight per sample) or encapsulated in HG containing an equivalent amount of GelMA (3 mg dry weight per sample). After culturing for 7 days, its viability was assessed using Calcein AM staining, an indicator of live cells due to its green fluorescence emission upon enzymatic conversion (Figure 2A).

Microscopic visualization revealed a strong green fluorescence along the surface of the HG, indicating robust cell viability in the outer regions (Figure 2A). However, within the central regions of the hydrogel (Figure 2A), fewer cells were observed, and their fluorescence intensity was lower, suggesting limited cell proliferation and potential viability constraints. This discrepancy is likely due to the diffusion limitations of nutrients and oxygen in the dense bulk hydrogel matrix.

Over time, hMSCs appeared to facilitate the aggregation of individual microparticles, possibly due to intercellular interactions between cells adhered to different MPs. Notably, fluorescence intensity was strongest along the periphery of these MP aggregates, as well as around individual microparticles (Figure 2A), indicating that surfaces of microparticles were still the preferred microenvironment for cell attachment and proliferation. To quantitatively compare cell densities in these two systems, we digested the samples using proteinase K to extract total DNA, a well-established method for assessing cell proliferation. The results (Figure 2B) demonstrated a significantly higher DNA content in cells cultured on MP than those encapsulated in HG, reinforcing the qualitative observations of increased cell growth on MP.

Beyond their proliferation capacity, hMSCs are known to secrete bioactive factors that contribute to wound healing by promoting cellular migration and tissue regeneration [15]. To assess this functional activity, we performed an in vitro scratch wound healing assay using human dermal fibroblasts (HDF). Conditioned media were collected from hMSCs cultured on MP and in HG, and their effects on HDF migration were evaluated by measuring the closure of an artificially created gap in a fibroblast monolayer over a 23-h period (Figure 2C).

The results showed that the wound gap closed more rapidly in the presence of conditioned media from hMSCs cultured on MP, indicating an increased secretion of pro-migratory factors. To quantitatively assess fibroblast migration, we measured the reduction in wound area using ImageJ software (Figure 2D). The analysis confirmed that conditioned media from MP cultures significantly enhanced fibroblast migration compared to media from hMSCs encapsulated in HG. This heightened migratory activity is likely due to the greater number of hMSCs present on MP than in HG and the efficient release of factors from hMSCs to the surrounding environment. To determine whether the composition of bioactive factors secreted by hMSCs differs between these two environments, a proteomic analysis would be beneficial.

### 2.3. Making Nanoparticles Containing Penicillin/Streptomycin

Because this crosslinking method takes place at physiological temperature and pH, cells and various biological molecules can be incorporated within the hydrogel without the danger of denaturing them. The hydrogel matrix may delay the release of hydrophilic bioactive molecules, preventing burst release and making it suitable for controlled delivery.

Since microparticles (MP) serve as more effective cell carriers for hMSCs than bulk hydrogels (HG), their potential as bioactive molecule carriers was evaluated. Gelatin nanoparticles (NP) have been used for drug and metal ion delivery due to their ability to stabilize cargos and prevent burst release [34]. Furthermore, bioactive molecule release from gelatin NP may be regulated through cell-mediated degradation under biological cues.

Delivering hMSCs via microparticles to wound environments holds therapeutic potential for wound care. Co-delivery of anti-infective treatments could further enhance efficacy. To explore if incorporating NP as an additional control of delivery would prolong the release of antibiotics and widen the therapeutic window, the antibiotics (penicillin/streptomycin (PS)) were incorporated into gelatin NP and compared with direct PS loading into hydrogel.

**Figure 3:**
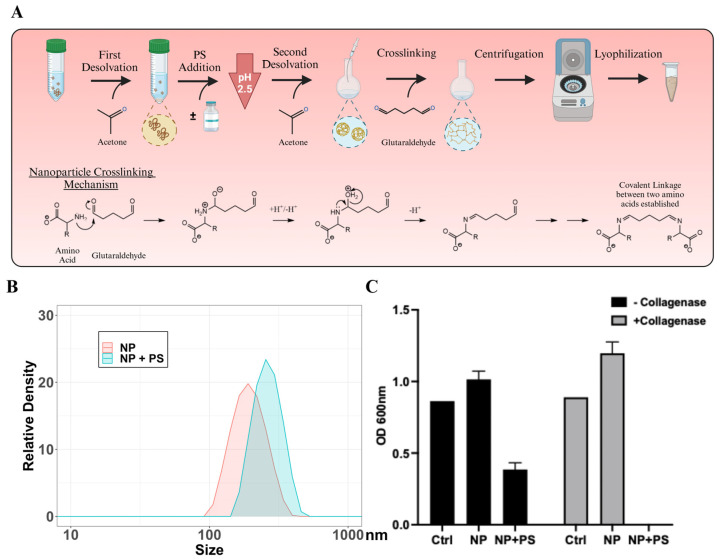
Characterization of gelatin nanoparticles (NP) containing penicillin/streptomycin (PS). (**A**) The key steps in synthesizing NP, with or without PS, are illustrated. (**B**) The size distribution of NP or NP+PS was determined using dynamic light scattering. (**C**) The absorbance at 600 nm (OD 600 nm) of *S. aureus* cultures in the presence or absence of NP or NP+PS was measured after 24 h incubation at 37 °C. The growth of *S. aureus* was measured with (gray) or without collagenase digestion (black).

To synthesize PS-loaded gelatin NP, the PS solution was added to gelatin and crosslinked with glutaraldehyde following an established method (Figure 3A). Both empty NP (referred to as NP) and PS-loaded NP (NP+PS) exhibited monodisperse characteristics (Figure 3B). Incorporation of PS slightly increased nanoparticle size from 184.9 ± 54.06 (PDI = 0.065) to 256.0 ± 61.12 (PDI = 0.020).

To evaluate antibacterial activity, *S. aureus* was cultured in the presence of NP or NP+PS (Figure 3C). NP+PS reduced *S. aureus* growth (black bars), and enzymatic digestion of NP+PS further enhanced antibacterial activity, eliminating all *S. aureus* (gray bars). These findings suggest that PS can be successfully incorporated into gelatin NP without losing its antibacterial efficacy during synthesis.

### 2.4. Making MPs Containing Various Configurations of NP

**Figure 4:**
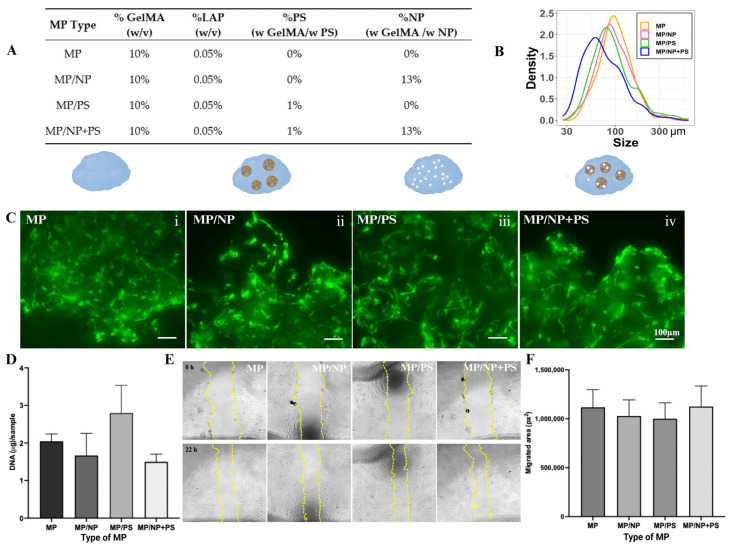
Characterization of four different configurations of MP. (**A**) The composition of different configurations of MP. (**B**) The size distribution of four configurations of MPs. (**C**) hMSCs were cultured on various MPs for 7 days and stained with CalceinAM. Representative projections of Z-stack images were shown. Scale bar =100 µm. (**D**) Total DNA from cells cultured on different MPs was extracted and quantified. (**E**) Representative wound images at 0 h (upper panel) and at 22 h (lower panel) under different conditions. The edge of the cell migration front was marked in yellow. (**F**) Quantification of the migrated area in the presence of conditioned medium from cells cultured on different MPs. Data shown are mean ± SD.

To assess whether loading NP or NP+PS into GelMA microparticles affects their function as carriers for hMSCs, the growth of hMSCs and migration of HDF were compared across different microparticle configurations (Figure 4).

Four types of microparticles were prepared (Figure 4A):

1. **MP (Plain GelMA):** GelMA hydrogel was micronized to produce microparticles;
2. **MP/NP:** Nanoparticles (NP) were incorporated into GelMA hydrogel before photo-crosslinking. The hydrogel was then cryo-micronized to produce NP-loaded microparticles;
3. **MP/NP+PS:** Nanoparticles containing PS (NP+PS) were incorporated into GelMA hydrogel, followed by photo-crosslinking and cryo-micronization to create NP+PS-loaded microparticles;
4. **MP/PS (Control):** A solution containing the same amount of PS, but without NP carriers, was added to GelMA hydrogel before microparticle formation.

The particle sizes of different MP configurations were measured, and their distributions are shown in Figure 4B. The size distributions of MP, MP/NP, and MP/PS were nearly identical, while MP/NP+PS exhibited a slight shift toward smaller sizes. The average particle sizes of MPs were 112.4 ± 64.5 µm for MP, 106.1 ± 50.9 µm for MP/NP, 107.0 ± 68.1 µm for MP/PS, and 85.8 ± 54.1 µm for MP/NP+PS.

hMSCs were seeded onto different MP types and cultured for 7 days. CalceinAM staining was performed to visualize viable cells (Figure 4C). The green fluorescence intensity was comparable across all MP configurations, indicating similar cell viability. Additionally, the spatial distribution of cells within MP aggregates was indistinguishable among the different carriers. To quantify cell growth on MP, samples were digested with proteinase K, and the extracted DNA was quantified (Figure 4D). No significant differences in DNA content were observed, suggesting that the incorporation of NP, PS, or NP+PS did not impair the ability of MP to support hMSC growth.

To further assess whether factors secreted by hMSCs grown on different MP configurations influenced HDF migration, conditioned media from hMSCs cultured on MP, MP/PS, MP/NP, or MP/NP+PS were applied to a scratch wound in an HDF monolayer. HDF migration, measured by wound closure (Figure 4E), was comparable across all conditions (Figure 4F).

These results indicate that incorporating nanoparticles or drugs does not compromise the functionality of MPs as cell carriers. Given the numerous successes of other BM-hMSCs treatment for chronic wounds, there is a high likelihood that these results will translate in vivo [35].

### 2.5. Antibacterial Activity of PS Carried by GelMA Hydrogel and GelMA MP

**Figure 5:**
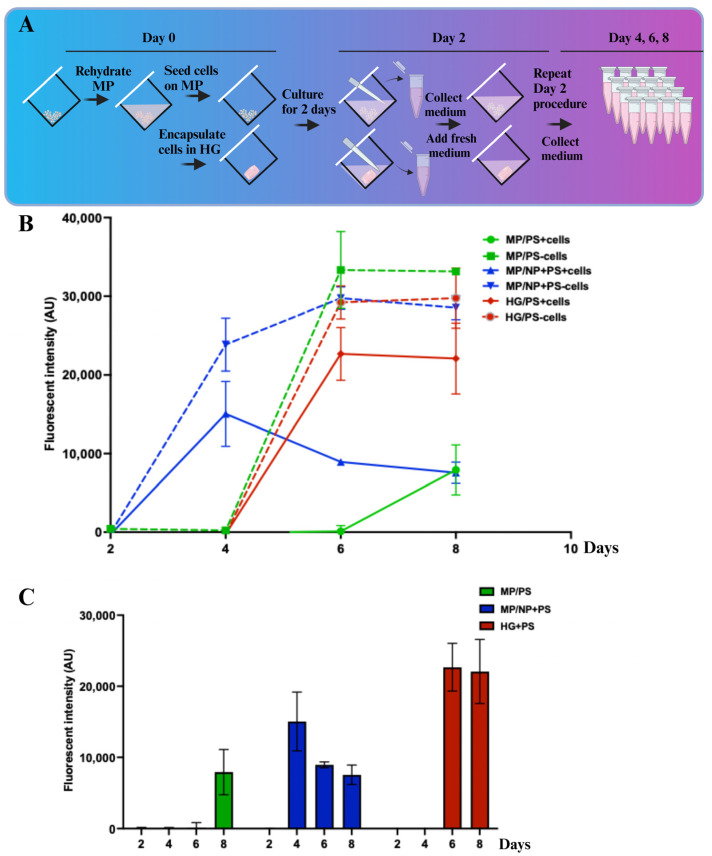
Release of anti-*S. aureus* activity from MPs or HG loaded with PS. (**A**) hMSCs were cultured on HG or various MP configurations. Culture medium from HG or MPs, with or without cells, was collected every two days and replaced with fresh medium. (**B**) The viability of *S. aureus* in the collected medium was assessed using the alamarBlue assay after 24 h. Dotted lines represent medium collected from samples without cells, while solid lines represent medium from samples with hMSCs. (**C**) *S. aureus* viability in medium collected on different days from MP/PS, MP/NP+PS, or HG/PS cultured with cells. These data are the same as in (**B**) but re-graphed to highlight samples with cells. Data are presented as mean ± SD.

MPs, as a cell carrier, were not negatively affected by carrying antibiotics (Figure 4). However, to be considered a viable carrier for drugs or antibiotics, the drug must be released from MP within a desired time window. In this experiment, the anti-*S. aureus* activity of PS in MP, MP/PS, and MP/NP+PS was compared with that of PS encapsulated in a GelMA hydrogel (HG/PS). The culture medium was collected every two days (on Days 2, 4, 6, and 8) (Figure 5A). Subsequently, 100 colony-forming units (CFU) of *S. aureus* were added to 100 µL of the collected medium (1 × 10^3^ CFU/mL). After 24 h of incubation, *S. aureus* viability was assessed using the alamarBlue assay (Figure 5B).

The medium collected on Day 2 from all MP configurations and HG effectively killed *S. aureus*, as no viable bacteria were recovered by colony numeration (data not shown). This suggests that a sufficient quantity of PS, exceeding the minimal bactericidal concentration (88 ng/mL streptomycin and 88 units/mL penicillin), was released from MP or HG. However, by Day 4, the PS released from MP/NP+PS was no longer sufficient to completely eliminate *S. aureus* (blue lines). Interestingly, in the presence of hMSCs, the medium from MP/NP+PS still exhibited inhibitory activity against *S. aureus* up to Day 8 (solid blue line). The striking difference between conditions with and without hMSCs suggests that cell-mediated degradation of gelatin NP may facilitate a sustained release of PS over time.

Anti-*S. aureus* activity was detected in the medium collected on Day 4 from HG/PS and MP/PS, indicating a continuous release of PS above its minimal inhibitory concentration (11 ng/mL streptomycin and 11 units/mL penicillin). However, by Day 6, anti-*S. aureus* activity was no longer detectable in HG/PS, whereas MP/PS covered with cells continued to release sufficient PS to inhibit *S. aureus*. Across all three PS carriers (MP/PS, MP/NP+PS, and HG/PS), the presence of hMSCs prolonged anti-*S. aureus* activity compared to the conditions without cells (compare dotted lines with solid lines). Two possible explanations for this observation are the following: (1) the cellular coverage of MP or HG surfaces acted as a physical barrier, reducing the initial burst release of PS, and (2) antimicrobial agents such as antimicrobial peptides secreted by hMSCs acted synergistically with low PS concentrations to inhibit *S. aureus*. Previous studies have shown that hMSCs produce antimicrobial peptides, which alone may not be sufficient to inhibit *S. aureus* but could complement the antimicrobial effect of antibiotics [36].

By Day 8, only the medium from MP/PS and MP/NP+PS cultured with cells showed inhibitory (but not bactericidal) effects against *S. aureus* (Figure 5B,C). This finding suggests that MP loaded with both cells and drugs may offer advantages in wound healing applications, making MP a promising candidate for the dual delivery of cells and therapeutics.

A limitation of this study is the lack of quantitative detection of PS release under different conditions. While we plan to develop high-performance liquid chromatography (HPLC) protocols to measure PS concentrations in solution, assessing anti-*S. aureus* activity remains a meaningful approach to evaluating the feasibility of dual delivery using MP.

### 2.6. Alternative Approach to Carry and Deliver NP

Nanoparticles have been widely used to prevent burst release and extend the release duration of therapeutic cargos [34]. In this study, NP+PS loaded within MP (MP/NP+PS) exhibited sustained PS release but at levels insufficient to eliminate *S. aureus*. In this formulation, PS was incorporated during synthesis and crosslinked with gelatin using glutaraldehyde. Although PS was released upon NP digestion by collagenase (Figure 3C), the gradual disintegration of NP may have been too slow for effective antibacterial activity.

**Figure 6:**
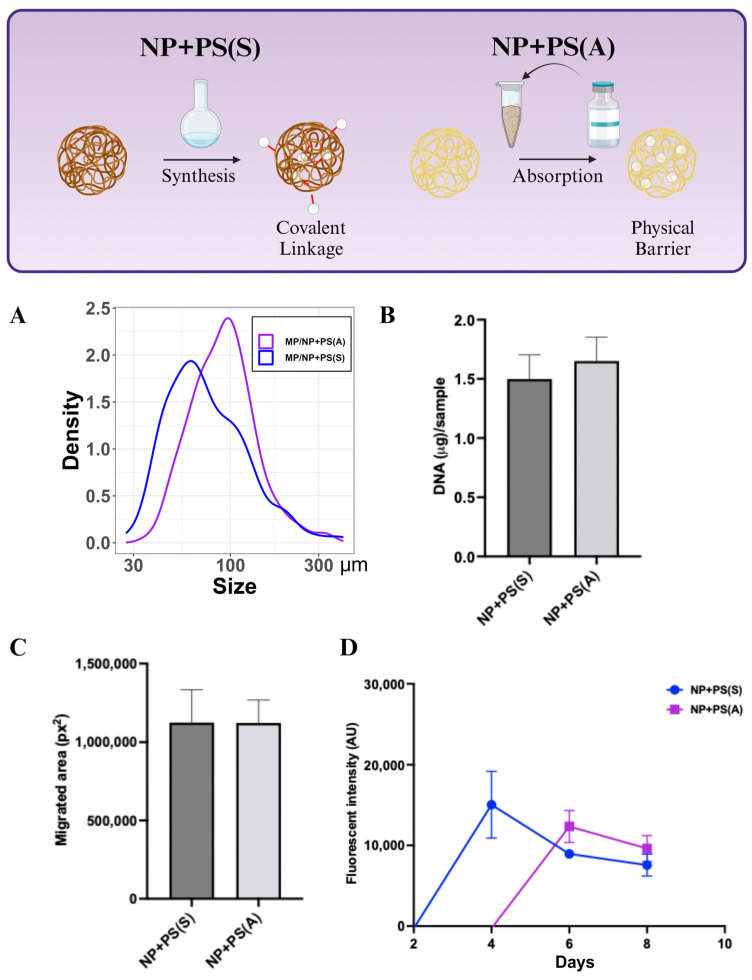
Comparison of MP/NP+PS(A) and MP/NP+PS(S). (**A**) The size distribution of the MP configurations. (**B**) Total DNA from cells cultured on both MP types were extracted and quantified. (**C**) Quantification of the migrated area in the presence of conditioned medium from cells cultured on different MP. (**D**) The viability of *S. aureus* in the collected medium was assessed using the alamarBlue assay after 24 h. Data shown are mean ± SD.

To test this hypothesis, an alternative loading method was explored: PS was absorbed into NP (NP+PS(A) indicating loading via absorption) rather than incorporated during synthesis (NP+PS(S) indicating loading via synthesis). Both types of NP-containing PS were then loaded in GelMA hydrogel and fabricated into MPs. Compared to MP/NP+PS(S), MP/NP+PS(A) exhibited a slightly larger size at 99.6 ± 46.2 µm compared to the former’s 85.8 ± 54.1 µm (Figure 6A). hMSCs cultured on these two types of MP showed no significant difference in total DNA content on Day 7 (Figure 6B). The conditioned medium from hMSCs cultured on MP/NP+PS(S) or MP/NP+PS(A) similarly promoted HDF migration (Figure 6C). These results indicate that PS loading via absorption did not impair the function of MP as a cell carrier.

When comparing the release of anti-*S. aureus* activity, MP/NP+PS (A) released sufficient PS to kill *S. aureus* on Day 4, whereas MP/NP+PS(S) did not. MP/NP+PS (A) continued to inhibit bacterial growth through Day 8, suggesting that absorption-based PS loading facilitates its release and may be a more effective and practical approach for PS-loaded NP fabrication. However, many hydrogels have been shown to take advantage of the wound environment to modulate the release of the loaded compound, so that drugs are only delivered when expressly required, and the rate of digestion of the scaffolds is one way to modulate release [37]. Since the wound bed contains high levels of matrix metalloproteinases due to neutrophils and certain bacteria, release profiles may differ as scaffolds are degraded, which may aid in releasing latent PS still trapped within the NP+PS (S) when needed.

Among all tested configurations, direct loading of PS into hydrogel before MP formation (MP/PS) demonstrated the strongest anti-*S. aureus* activity, sustaining efficacy for up to 8 days. Since it typically requires 3–5 days to establish a stable hMSC culture in vitro, antibacterial activity released within this timeframe may not be fully utilized in a wound environment. A delayed release, such as after Day 4, could be more relevant for combating infection in wounds. To confirm whether MP/NP+PS provides longer-lasting PS release than MP/PS, extended quantification of PS release over time using HPLC will be necessary. PS served as a model cargo to explore the potential of MP for dual delivery. Given the mild MP fabrication process, other bioactive molecules, such as peptides and growth factors, could also be co-delivered with cells for regenerative applications. To determine the therapeutic potential of MP as a dual-delivery system for hMSCs and antibiotics, further evaluation in preclinical wound healing models is warranted.

**Figure 7:**
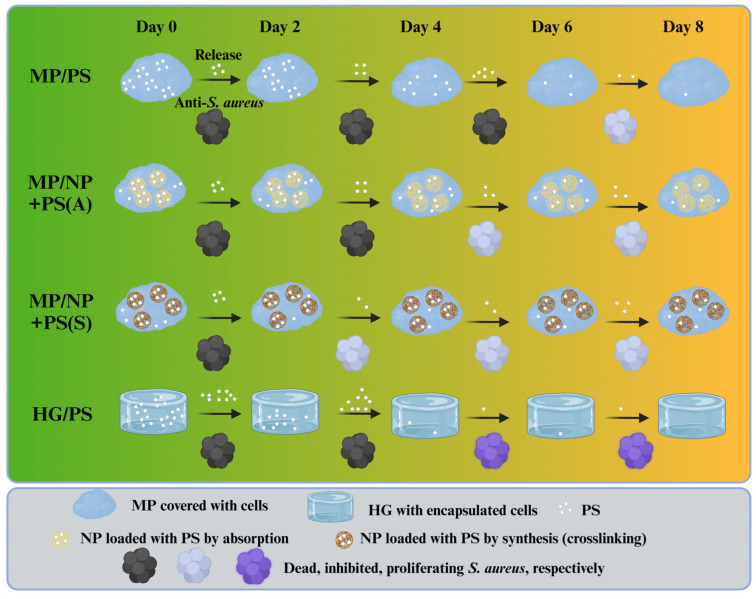
The anti-*S. aureus* activity released from MPs or HG was postulated and summarized.

To account for the observed anti-*S. aureus* activity across different delivery configurations, Figure 7 illustrates the proposed PS release and *S. aureus* inhibition mechanisms. MP/NP+PS (S) completely killed the bacteria for up to two days of culture and inhibited growth up until day eight. MP/NP+PS (A) and HG/PS could eliminate bacteria for up to four days of culture. However, HG/PS showed no effect after the four-day mark, while MP/NP+PS (A) inhibited growth until day eight. MP/PS was able to kill bacteria for the longest period of time, up to six days, and it inhibited growth until day eight.

## 3. Materials and Methods

### 3.1. Making GelMA Microparticles (GelMA MP)

Synthesis and characterization of methacrylated gelatin (GelMA) was conducted as previously described [16,38]. The GelMA MP was prepared as previously described [39]. Briefly, the photoinitiator lithium phenyl-2,4,6-trimethylbenzoylphosphinate (LAP, Sigma Aldrich, St. Louis, MO, USA) at a final working concentration of 0.05% (*w/v*) was added to a 10% GelMA solution and mixed. The mixture was transferred to wells of a polydimethylsiloxane (PDMS)-coated, 6-well plate (1.25 mL/well). The GelMA solutions in the wells were then crosslinked with light (405 nm) (Sovol, Shenzhen, China) at 20 mW cm^−2^ and at a distance of 13 cm from the top of the well for 3 min. The GelMA gels were then flipped, and light crosslinking was repeated for the other side. The crosslinked hydrogels were then cut into approximately 3 mm × 3 mm pieces and frozen at −80 °C or in liquid nitrogen. Once completely frozen, the GelMA pieces were transferred into a pre-chilled mortar and pestle and broken down into finer pieces using a repeated grinding motion. The morphologies of the microparticles were sampled and examined under a light microscope. If necessary, the sample in mortar was placed back into −80 °C to ensure the samples remained frozen. When the majority of the particle sizes reached about 100 µm, the samples were suspended in 1.2 mL of diH_2_O, frozen at −80 °C, lyophilized, and then stored at 4 °C until use.

### 3.2. Synthesis of Nanoparticles with or Without Penicillin-Streptomycin

Synthesis of nanoparticles (NPs) was carried out following the previously reported procedure [40]. Briefly, 500 mg of porcine gelatin (Sigma-Aldrich, Burlington, MA, USA) was dissolved in 10 mL of diH_2_O at 40 °C. Once fully dissolved, 10 mL of acetone (Sigma-Aldrich, St. Louis, MO, USA) was added gently, and the solution was swirled until homogenous. The solution was incubated at room temperature for 1 h to allow the high molecular weight (HMW) gelatin to precipitate. The precipitated HMW gelatin was washed twice with 2 mL of diH_2_O. A total of 10 mL of diH_2_O or 9 mL of diH_2_O + 1 mL of penicillin-streptomycin (PS) stock (10 mg/mL penicillin and 10,000 units of penicillin) (ThermoFisher, Branchburg, NJ, USA) was added to the washed gelatin and incubated at 40 °C to dissolve and mix. Once fully dissolved, the pH was adjusted to 2.5 using 1 M HCl. A total of 30 mL of acetone was added at a rate of 3 mL/min to the mixture. Immediately after adding all of the acetone, 140 µL of 25% glutaraldehyde solution (Sigma-Aldrich, St. Louis, MO, USA) was added. The solutions were mixed at 40 °C for 1 h and at room temperature for 18 h. The NP+PS solution was passed through a 0.22 µm filter to remove any large particles. The reaction mixtures were then centrifuged at 15,000× *g* for 20 min. All samples were then dispersed in 1 mL of diH_2_O by ultrasonication and then frozen at 80 °C. The lyophilized NPs were stored at 4 °C till use.

### 3.3. Loading Nanoparticles Using the Absorption Method

A total of 16.8 mg of empty NP were sterilized under UV light for 30 min. In all, 82.9 µL of stock PS (10 mg/mL streptomycin and 10,000 units/mL penicillin) was added and mixed to create a slurry. The sample was air-dried for 18 h before incorporation into MP.

### 3.4. Particle Size Analysis of Nanoparticles

To determine the size of the nanoparticles, empty NP (referred to as NP) and antibiotics-loaded NP (referred as NP+PS) were rehydrated in diH_2_O at room temperature for 1 h. NP solutions were then dispersed by ultrasonication. One mL of dispersed NPs was added to a cuvette and analyzed using Zetasizer Nano Series (Malvern Instruments, Worcestershire, UK). The particle size and distribution were analyzed using Zetasizer Nano Series (Nano-S) Software.

### 3.5. Making GelMA Microparticles (GelMA MP) Containing PS

Various formulations were used to make GelMA hydrogels containing NP, NP/PS, and PS solution (Table 1). In regards to cryo-milling microparticles, for each condition, the following solution was prepared.

For MP/NP+PS(A) and MP/NP+PS(S), 600 µL of the total PBS was used to dissolve the NPs followed by ultrasonication to ensure dispersion before addition to the final solution.

A total of 1.25 mL of each solution was added to a PDMS-coated, 6-well plate. The MP with various formulations was prepared as described in Section 3.5.

### 3.6. Culturing Human Mesenchymal Stem Cells

Human bone marrow mesenchymal stem cells (hMSCs) were purchased from Texas A&M University (Lot 8011L, College Station, Brazos County, TX, USA). Passages 3–6 of hMSCs were used for this study. Cells were cultured in MEM-alpha medium (HyClone, Logan, Cache County, UT, USA) supplemented with 10% fetal bovine serum (CPS Serum, Kansas City, MO, USA) and 25 mg/mL of gentamicin (Sigma-Aldrich, Burlington, MA, USA) in tissue culture-treated polystyrene plates (CELLTREAT, Pepperell, MA, USA). The plates were incubated in a tissue culture incubator (Panasonic, Wood Dale, DuPage County, IL, USA) at 37 °C, 5% CO_2_, and 95% humidity. When the cell density reached about 80% confluence, cells were washed with PBS, trypsinized, counted, and resuspended in assay medium (growth medium without antibiotics) to be seeded as described below.

### 3.7. Seeding hMSCs on Cell Carriers

A total of 3 mg/sample of microparticles in various configurations were weighed out and sterilized under UV for 30 min. Microparticles were rehydrated in a 0.5 mL assay media for 1 h and spun down at 12,300× *g* for 5 min, and the rehydration media was removed. A total of 100 µL of assay media containing 6–8 × 10^4^ hMSCs was added to each sample. All samples were placed in the incubator for 30 min to allow cells to attach to the microparticles and were gently mixed by tapping every 5 min. The samples were then transferred to a 48-well cell-repellant plate (Greiner Bio-One, Kremsmünster, Austria), and 0.3 mL of the assay media was then added to each well and incubated in a tissue culture incubator. The plate was incubated in the tissue culture incubator, and the assay media were changed every two days.

To encapsulate hMSCs in GelMA, GelMA (3 mg dry weight/sample) was dissolved in an assay medium to make a 10% solution. For HG/PS, the PS solution (10 mg/mL streptomycin + 10,000 units of penicillin) was added for a final concentration of 0.82 mg/mL streptomycin and 8200 units penicillin/mL. Afterward, 6–8 × 10^4^ hMSCs/sample was added to the GelMA solution, followed by the photoinitiator LAP for a final concentration of 0.5 mg/mL. A total of 30 µL of the solution was pipetted into each well (5 mm in diameter × 1 mm in thickness) of a PDMS mold. The samples were crosslinked under a 405 nm UV lamp for 40 s. The discs of the GelMA hydrogel (GelMA HG) were gently removed from the PDMS mold and transferred to a 48-well cell-repellant plate. After washing with 0.4 mL of PBS, the GelMA HG with or without cells were then incubated at 0.4 mL/sample assay media at 37 °C. At the end of cell culturing, the quantity of cells on carriers were determined by extracting and quantifying DNA from each sample as described previously [41].

### 3.8. Preparation of Samples for Antimicrobial Testing

#### 3.8.1. Preparation of Samples from Nanoparticles

A total of 6 mg of NP and NP+PS(S) were weighed and sterilized under UV for 30 min. The samples were dispersed in 600 µL of PBS by ultrasonication, rehydrated for 1 h, and ultrasonicated once more. The samples were split evenly into six Eppendorf tubes (100 µL/tube). A total of 100 µL of 2 mg/mL of collagenase (Worthington Biochemical, Lakewood, NJ, USA) was added to half of the samples, and 100 µL of PBS to the other half. The samples were incubated for 2 h at 37 °C.

#### 3.8.2. Preparation of Samples from Various Configurations of Microparticles and GelMA HG

Cell carriers (microparticles or GelMA HG) seeded with cells or carriers alone were incubated in assay medium (Mem-alpha + 10%FBS) in a 48-well cell repellent plate. To test the antimicrobial activity released from cell carriers or cell carriers with cells, the conditioned medium from each well was collected every two days.

### 3.9. Antimicrobial Testing

#### 3.9.1. Antibacterial Activity of NP Containing PS

A total of 200 µL of 1 × 10^6^ CFU/mL of *Staphylococcus aureus* (ATCC 25923™ American Type Culture Collection, Manassas, VA, USA) was added to each sample (200 µL) and incubated for 18 h. The optical densities of the overnight cultures were then measured at 600 nm.

#### 3.9.2. Antibacterial Activity of Various Configurations of HG or MP

Samples were collected as described in Section 3.8.2. A total of 100 µL/well of samples were added to a 96-well plate. A total of 10 µL of *S. aureus* (2 × 10^5^ colony formation units, CFU) in tryptic soy broth (Sigma-Aldrich) were added to each sample. The plate was then incubated at 37 °C overnight. To determine the viability of bacteria, 10 µL of alamarBlue reagent (Bio-Rad, Hercules, CA, USA) was added to each sample in the 96-well plate. The plate was then incubated for 1 h at 37 °C in the dark. The fluorescence intensity was read at Ex/Em = 540 nm/590 nm using a TECAN Spark (Spark^®^, TECAN, Mendrisio, Switzerland).

### 3.10. Cell Viability Staining by Calcein Acetoxymethyl Ester (CalceinAM)

hMSCs were cultured on various microparticles or in GelMA HG for 7 days. A total of one μM of CalceinAM (Corning Inc., Corning, NY, USA) was added to samples in assay medium. After incubating for 30 min at 37 °C and washing with PBS, samples were examined using a fluorescence microscope (Echo Revolve, San Diego, CA, USA).

### 3.11. Scratch Wound Migration Assay

hMSCs were cultured on different microparticles or hydrogel discs in assay medium for 7 days. To prepare conditioned media for the cell migration assay, 0.45 mL/well of MEM-alpha base medium (assay medium without FBS) was added to each well. The cultures were incubated at 37 °C with 5% CO_2_ and 95% humidity for 24 h. The supernatants (conditioned media) were collected from each well and used in the scratch migration assay. The migration assay was performed as described previously [16]. Briefly, human dermal fibroblasts (HDF, ATCC) were cultured to 100% confluency in a 48-well TCP plate. A scratch wound was made on the cell monolayer in each well. The scratch wounds were marked and imaged, and 0.2 mL of conditioned media was added to each well. After 23 h, the exact same wound areas (with marker references) were imaged again. Wound areas were measured using ImageJ software (version: 2.14.0/1.54f, NIH) (square pixels, px^2^). The migrated area was calculated as follows:

Migrated area = Area_0h_ − Area_23h_.

### 3.12. Statistical Analysis

For each experiment, at least three biological repeats were used (*n* ≥ 3), and data are presented as mean ± standard deviation. Statistical analysis was performed as previously described [42]. One-way ANOVA with a Tukey’s multiple comparisons test was performed to determine statistical significance using GraphPad Prism version 10.4.1 (accessed on 2 January 2025 GraphPad Software) (La Jolla, CA, USA, www.graphpad.com) for all quantitative data. Differences were considered significant at a *p*-value of <0.05.

## 4. Conclusions

In this study, we developed an advanced dual-delivery system capable of simultaneously delivering stem cell therapy and anti-infection treatment. Building upon the widely used hydrogel encapsulation approach for stem cell delivery, we designed micronized hydrogel microparticles (MP) to create an optimal environment for human mesenchymal stem cells (hMSCs) to enhance their delivery. To modulate the release of antibiotics (PS) from these cell carriers, we explored multiple strategies, including the incorporation of gelatin nanoparticles. Interestingly, we observed a striking synergistic effect between stem cells and antibiotics, highlighting the potential of this dual-delivery system in treating chronic wounds. Furthermore, the gelatin nanoparticle-assisted delivery of PS may leverage the protease-rich wound environment to enable a need-based drug release. The demonstrated feasibility of this system paves the way for co-delivering bioactive molecules alongside cells, extending its regenerative applications beyond wound healing.

## Figures and Tables

**Table 1:** Components of the solution used to make each MP type. Each column represents components that were added directly to solution. For MP/NP+PS(A) and MP/NP+PS(S), antibiotics were loaded beforehand in NP, which is why they were heavier than NP added to MP/NP. NP+PS(S) were assumed to consist of 8.11% antibiotics by weight. This was calculated by assuming that all the mass of the antibiotics added entered into the NPs. The quantities of NPs and antibiotics added were chosen so that the weight of antibiotics would make up 1% of the dry weight of the MPs.

MP Type	GelMA (mg)	PBS (µL)	PS (µL) (10 mg/mL Strep + 10,000 Units Penicillin)	LAP (µL) (10 mg/mL)	NP (mg)
MP	130	1235	0	65	0
MP/NP	130	1235	0	65	16.8
MP/PS	130	1235	82	65	0
MP/NP+PS(A)	130	1235	0	65	18.3
MP/NP+PS(S)	130	1235	0	65	18.3

## Data Availability

Due to an ongoing related project, all data are available upon request.

## Abbreviations

The following abbreviations are used in this manuscript:

BM-hMSC or hMSC	Bone marrow-derived human mesenchymal stem cells
HG	Hydrogel
HG/PS	Hydrogel with antibiotics directly embedded
MP	Microparticles
MP/NP	Microparticles embedded with empty nanoparticles
MP/PS	Microparticles with antibiotics directly embedded
MP/NP+PS or MP/NP+PS(S)	Microparticles embedded with nanoparticles containing antibiotics loaded via synthesis
MP/NP+PS(A)	Microparticles embedded with nanoparticles containing antibiotics loaded via absorption
